# Metacontrol instructions lead to adult-like event segmentation in adolescents

**DOI:** 10.1016/j.dcn.2025.101521

**Published:** 2025-01-30

**Authors:** Xianzhen Zhou, Foroogh Ghorbani, Veit Roessner, Bernhard Hommel, Astrid Prochnow, Christian Beste

**Affiliations:** a Cognitive Neurophysiology, Department of Child and Adolescent Psychiatry, Faculty of Medicine, TU Dresden, Schubertstrasse 42, Dresden 01307, Germany; b German Center for Child and Adolescent Health (DZKJ), Partner site Leipzig/Dresden, Dresden, Germany; c School of Psychology, Shandong Normal University, Jinan, China

**Keywords:** Event segmentation, Development, Metacontrol, EEG

## Abstract

Event segmentation, which involves dividing continuous information into meaningful units, changes as children develop into adolescents. Adolescents tend to segment events more coarsely than adults. This study explores whether adolescents could adjust their segmentation style to resemble that of adults when provided with explicit metacontrol-related instructions. We compared event segmentation in two adolescent groups and one adult group, while simultaneously recording EEG data. One adolescent group was instructed to perform segmentation as finely as possible, whereas the other adolescent group and adults received no specific instructions on segmentation granularity. EEG data were analyzed using multivariate pattern analysis and source reconstruction. The findings revealed that adolescents given fine-grained instructions adjusted their segmentation probability closer to adult levels, although they did not fully match adults in processing multiple simultaneous changes. Neurophysiological results indicated that adolescents with fine-grained instructions exhibited neural decoding performance more similar to adults. Increased activity in the inferior frontal gyrus in these adolescents compared to adults related to this. The results suggest that adolescents with fine-grained instructions demonstrated more persistent cognitive control and enhanced top-down attention than their peers and adults. The study shows that adolescent cognitive processes can be shifted toward adult-like performance through instructions.

## Highlight

• Cognitive processes in adolescents can be shifted to adult-like performance just by instruction.
• EEG showed adolescents with fine-grained instructions had neural patterns more similar to adults.
• Increased activity in the inferior frontal gyrus contributes to their more adult-like behavior.

## Graphical Abstract

**Figure:**
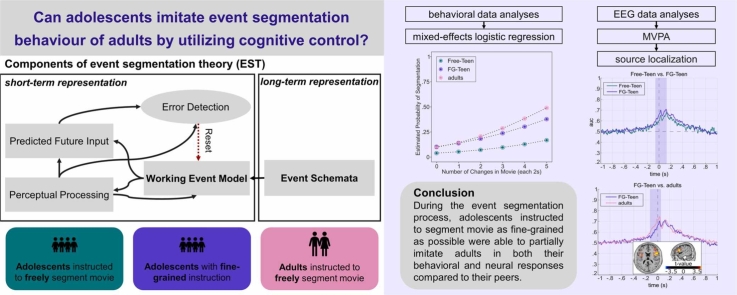

## 1 Introduction

“Please behave yourself in a more mature way” – likely belongs to the most common expressions of parents. However, the question of how much children and adolescents can adjust their behavior towards an adult-like level and what neural processes are called on to support this ability still remains unanswered. One fundamental cognitive function that may be key to this is the ability to segment incoming information into meaningful segments (Radvansky and Zacks, 2014, Zacks, 2019), which likely reflects an important basis for goal-directed action. This ability undergoes maturational changes (Benear et al., 2023, Ghorbani et al., 2024, Ren et al., 2021). According to the Event Segmentation Theory (EST, Zacks et al., 2007), two types of event representations are important in event segmentation: First, there are working event models, actively held in working memory, depicting ongoing activities. They set the basis for predicting how events are most likely to evolve. Second, there are event schemata, representations of various types of events stored in long-term memory and used to create the event models (Zacks, 2019). The ability to integrate various information into a working event model is correlated with working memory capacity (Radvansky and Copeland, 2006), which increases during cognitive development (Cowan, 2016, Spencer, 2020). Moreover, due to their limited life experience, children and adolescents typically have fewer event schemata available than adults, resulting in a restricted and superficial understanding of different event types (Levine et al., 2019).

The developmental process of event segmentation is closely linked with the hierarchical organization of event segmentation, as previous studies have shown that children and adolescents are more likely to segment incoming information in coarser bins of events than adults (Ghorbani et al., 2024, Meyer and Baldwin, 2011, Yates et al., 2022). However, most research has been conducted in young children and even infants (Benear et al., 2023, Chai et al., 2024, Yates et al., 2022); thus, event segmentation in adolescents is not fully understood. In addition, it is known that adults can purposefully segment activities into different levels of granularity (Zacks et al., 2009, Zacks et al., 2001), requiring them to adapt cognitive control strategies to varying levels of detail (Kurby and Zacks, 2008, Zacks and Tversky, 2001). Adjusting the granularity of segmented events can be seen as an effect of metacontrol (Zhou et al., 2024). Metacontrol allows individuals to balance persistent and flexible cognitive control styles, directing information processing towards either a more detailed or a broader focus (Hommel, 2015a, Hommel et al., 2024, Hommel and Colzato, 2017a, Van Schependom et al., 2024, Wang et al., 2024). In a rather persistence-heavy processing style characterized by robust top-down cognitive control and a narrow focus, finer-grained events are formed. We hypothesize that adolescents might also be able to adapt their segmentation style with different instructions. When wanting adolescents to behave in a more adult-like way, they should thus resemble a more persistence-heavy processing style and, therefore, be instructed to find finer-grained segments. In the current study, we used metacontrol manipulations to examine the neurophysiological principles underlying how adolescents can behave in a more adult-like way.

One instruction asked the participants to segment a movie in a meaningful manner when watching it, while another instruction was added to this meaningful segmentation instruction to be as fine-grained as possible. We expected adolescents instructed to perform a rather fine-grained segmentation to exhibit outcomes more akin to those of adults than their peers without such specific instruction. Given that both the working event models and event schemata could be considered as mental representations undergoing development throughout childhood and adolescence (Kurby and Zacks, 2008, Zacks and Swallow, 2007), we assumed that the neural representation patterns of the adolescents instructed to segment as fine-grained as possible would closely resemble those of adults compared to their peers. A method that was suitable to precisely capture the dynamics of these neural representation patterns during the event segmentation process was the multivariate pattern analysis (MVPA, (Fahrenfort et al., 2018, Grootswagers et al., 2017, King and Dehaene, 2014, Petruo et al., 2021, Takacs et al., 2020) on EEG recordings. Specifically, temporal generalization MVPA was employed for its ability to generalize neural patterns from one time point to another, providing insight into the stability and reactivation of event representations over time. Furthermore, source reconstruction methods were utilized to examine which brain regions were associated with the differences between adolescents’ and adults’ neural representation patterns (sLORETA, Pascual-Marqui, 2002). Adolescents instructed to segment as fine-grained as possible were expected to focus more on detailed changes in the movie by employing a more persistent cognitive control style than their peers. Consequently, their working event models would compare predictions and inputs more frequently, necessitating increased online monitoring. Therefore, we hypothesized that in adolescents instructed to segment the movie in a fine-grained manner, MVPA outcomes would be better decodable than in their peers, particularly during the time window surrounding boundaries of events. Additionally, during this period, it was assumed that they would show heightened activity in brain regions associated with performance monitoring, such as fronto-polar regions (Koechlin, 2014, Mansouri et al., 2017), insula cortex (Gogolla, 2017, Ham et al., 2013) and anterior cingulate cortex (Carter et al., 1998, Ham et al., 2013).

## 2 Materials and methods

### 2.1 Sample

The sample consisted of three groups of participants. There were two adolescent groups (age range 11–16 years), one with a fine-grained segmentation instruction (FG-Teen, N = 42) and an age- and gender-matched group with a free segmentation instruction (Free-Teen, N = 42, see Task for details). Moreover, there was an adult group (age range 20–30 years) receiving the free segmentation instruction (N = 45). The participants were recruited via an in-house database and advertisements. Before participation, the participants and/or their legal guardians underwent a brief telephone interview to exclude any psychiatric or neurological conditions. After the data collection was conducted, there were exclusions from the final analysis due to poor EEG data quality (N = 3 Free-Teen, N = 3 FG-Teen, N = 2 adults) and too few key presses for a behavioral analysis. After within-group outlier exclusions regarding the number of key presses (N = 1 Free-Teen, N = 2 FG-Teen, N = 2 adults) and the mean segment length (N = 2 Free-Teen, N = 6 FG-Teen, N = 3 adults), the final sample consisted of N = 34 Free-Teen (14.06 ± 1.45 years, 12 males), N = 32 FG-Teen (14.19 ± 1.57 years, 10 males) and N = 36 adults (26.06 ± 2.77 years, 18 males). The participants and, if applicable, their legal guardians provided written informed consent before the participation in the study. The local ethics committee approved the study.

### 2.2 Task

The participants completed an event segmentation task while watching the short movie “The Red Balloon” (Le ballon rouge, 1956), which has frequently been used in event segmentation tasks (e.g., Prochnow et al., 2024c; Zacks et al., 2009; Zacks, 2010) and recently implemented for EEG research (Ghorbani et al., 2024, Prochnow et al., 2024c, Prochnow et al., 2024b). In the free segmentation instruction, the participants received the instruction to “press the space key whenever something in the movie ended, and something else was about to start,” while in the fine-grained segmentation instruction, the participants were told to “define these units as small as possible.” For the adult sample, the movie was separated into four clips (durations of 7:43 min, 7:48 min, 7:26 min, and 10:00 min), whereas for the adolescent sample, the movie was separated into three clips (durations of 10:22 min, 11:08 min, 11:05 min) to create the illusion of the total task duration being shorter and thus increase the commitment (Ghorbani et al., 2024). Between the clips, all participants could take breaks of self-chosen length. Before conducting the event segmentation task on “The Red Balloon”, the participants conducted a supervised exercise to ensure they understood the task instruction. To this end, different short videos (about 5 min duration) were chosen for practicing which have been used previously in event segmentation research (Bailey et al., 2013, Sargent et al., 2013). The situational change coding for “The Red Balloon” established by Zacks et al. (2009) was used to analyze the segmentation behavior in the task.

### 2.3 EEG recording and preprocessing

During the event segmentation task, EEG signals were recorded using elastic caps (EasyCap Inc.) outfitted with 60 Ag/AgCl electrodes, with the reference electrode placed at Fpz and the ground electrode at θ = 58, ϕ = 78. EEG signals were amplified using BrainAmp amplifiers (Brain Products Inc.), while ensuring electrode impedances remained below 5 kΩ. A sampling rate of 500 Hz was employed online, which was subsequently down-sampled to 300 Hz during offline preprocessing. Data preprocessing was conducted using the "Automagic" pipeline (Pedroni et al., 2019) and EEGLAB (Delorme and Makeig, 2004). Initially, flat channels were identified and removed, followed by applying an average referencing to the EEG data. Subsequently, the PREP preprocessing pipeline and the EEGLAB 'clean_rawdata()' pipeline were applied. Line noise at 50 Hz was eliminated, and a robust average reference was employed post-removal of contamination by bad channels. A finite impulse response (FIR) high-pass filter (.5 Hz, order 1286, stop-band attenuation −80 dB, transition band.25–.75 Hz) was utilized to detect and exclude flat-lined, noisy, or outlier channels. To remove electromyographic (EMG) artifacts, a low-pass filter of 40 Hz (sinc FIR filter; order: 86;(Widmann et al., 2015) was applied. Electro-oculographic (EOG) artifacts were discarded using a subtraction method (Parra et al., 2005). Muscle, cardiac, and residual ocular artifacts were addressed via Independent Component Analysis (ICA) utilizing the Multiple Artifact Rejection Algorithm (MARA; (Winkler et al., 2014). Artifact Subspace Reconstruction (ASR; burst criterion: 15; (Mullen et al., 2013) was employed to reconstruct epochs with abnormally strong power (>15 standard deviations relative to calibration data) within the segmented data (details provided below). Time windows that could not be reconstructed were discarded. Finally, any missing or eliminated channels were interpolated using a spherical method.

**Fig. 1:**
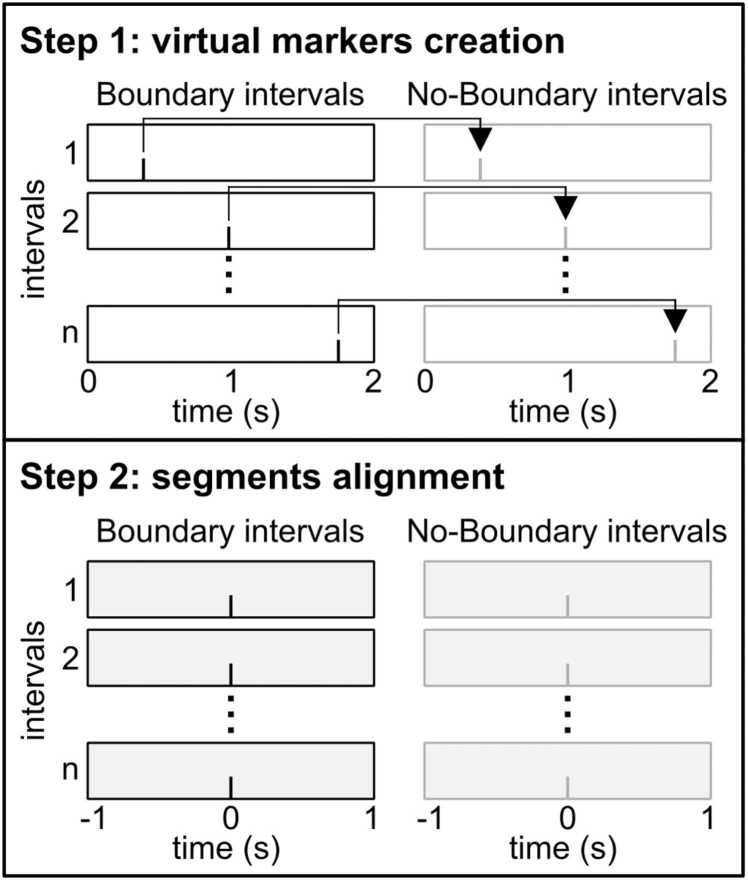
Schematic illustration of the creation of virtual markers for No-Boundary intervals. Boundary intervals are indicated in black, No-Boundary intervals are shown in grey. In the first step, Boundary intervals were randomly assigned to No-Boundary intervals. Virtual markers were then placed within the No-Boundary intervals at the same time point as the key press in the corresponding Boundary interval (upper section). In the second step, data were re-segmented according to these markers, allowing for the analysis of data from −1–1 sec relative to the marker's position (lower section).

We utilized FieldTrip (Oostenveld et al., 2011) for subsequent analysis procedures. To elucidate the distinction between time windows containing event boundaries and those lacking such boundaries, we delineated Boundary intervals (BI; characterized by key presses indicating event boundaries) and No-Boundary intervals (NBI; without key presses indicating event boundaries), following the protocol outlined by Prochnow et al. (2024c). While BI inherently encompassed response markers (key presses), NBI lacked such markers. Thus, we inserted virtual markers for NBI based on response markers by implementing the following steps (Fig. 1): (a) Aligning with behavioral data, continuous data were segmented into 2 s intervals. Notably, there were more intervals lacking response markers than those containing them across all participants. (b) For each participant, intervals without response markers were randomly chosen in a quantity matching that of intervals with response markers. These intervals were then randomly paired with intervals possessing response markers. (c) The time instance of a response marker within an interval was projected as a virtual marker onto the corresponding interval without a response marker, as assigned in step (b). In doing so, corresponding response markers for BI and virtual markers for NBI were created, maintaining consistent numbers across participants. This study adopted response-locked data analysis for two principal reasons: Firstly, the absence of distinctly separable stimuli akin to conventional EEG paradigms, and secondly, the pivotal nature of event segmentation, as delineated by the timing of motor responses or key presses. Subsequently, data spanning from -1s to 1 s relative to both types of markers were included in subsequent analysis stages.

### 2.4 Multivariate pattern analysis (MVPA)

To discern differences between BI and NBI over time, we employed multivariate pattern analysis (MVPA) on time domain data utilizing the MVPA-Light toolbox (Treder, 2020) as done in previous studies by our group (Ghin et al., 2024, Graf et al., 2024, Prochnow et al., 2024a, Yu et al., 2023). Each individual subject underwent two distinct analyses. Within each trial, only signals within the −1–1 s timeframe relative to key presses were fed into the MVPA. First, binary classification across time was conducted to identify specific time points showing distinctive spatial patterns on the electrode level between BI and NBI. Second, to investigate the temporal dynamics of representational content, temporal generalization MVPA was performed. The classifier utilized in this study was Regularized Linear Discriminant Analysis (Renton et al., 2022) evaluated via a 10-fold cross-validation approach repeated 10 times. Default parameter values in the MVPA-Light toolbox were employed, unless stated otherwise. Classification accuracy was assessed using the area under the curve (AUC), a non-parametric measure derived from signal detection theory, and the classification accuracy. Within each group, time points exhibiting significant classification performance, as indicated by AUC values, were identified through cluster-based permutation testing. This entailed 1000 random draws and employed non-parametric Wilcoxon tests with a significance level of p = .05. The cluster level statistic was determined by summing all Wilcoxon test values within the specified time range. The null value for AUC was set at a chance level of 0.5, corresponding to 50 %. To conduct statistical comparisons of the MVPA results between the groups, cluster-based permutation tests were employed to compare the outcomes of different MVPA analyses. The Matlab function *permutest* (Gerber, 2022) was applied for these comparisons by utilizing independent t-tests between two groups. A reference distribution was generated through 1000 random draws, with an alpha value of p = .001 set for the t-tests. Each significant cluster's direction of effect (positive or negative) and the sum of t-values within the cluster (T_sum_) are provided.

### 2.5 Source localization (sLORETA)

Subsequently, we employed standardized low-resolution brain electromagnetic tomography (sLORETA, (Pascual-Marqui, 2002) identify the neuroanatomical structures contributing to the different neural representation patterns detected for various groups through MVPA. Specifically, we selected the significant time windows from the MVPA results for each pair of groups to determine which brain regions were involved in these differences during this time window. During these time windows the difference between BI and NBI within a group was contrasted with the same difference in the comparison group. sLORETA utilizes a realistic MNI152 head model, dividing the intracerebral volume into 6239 voxels with a spatial resolution of 5 mm, and subsequently calculates a standardized current density for each voxel (Pascual-Marqui, 2002). This method offers a linear solution to the inverse problem while avoiding localization bias (Sekihara et al., 2005), and the reliability of this method has been corroborated by brain stimulation and MRI studies (Dippel and Beste, 2015, Ocklenburg et al., 2018). We performed a built-in voxel-wise randomization test with 2000 permutations to assess statistical significance, employing statistical non-parametric mapping (SnPM). The results section presents voxels in the MNI brain template showing significant differences (p < 0.05) in the modulation between each pair of groups.

### 2.6 Statistics

A mixed-effects logistic regression model was utilized to analyze behavioral effects. To this end, the movie was divided into time bins of 2 sec. The predictor was the number of situational changes within each 2-second interval (ranging from 0 to 5), while the outcome variable was the occurrence of a response within this interval (0 indicating no response, 1 indicating a response occurred). Additionally, group membership was incorporated as a predictor in the model, always comparing two of the groups with each other. Random intercepts for subjects were estimated in both models to account for variability between subjects. Odds ratios (ORs) were computed from the coefficient results of the fixed effects in both models, and multicollinearity was assessed using the variance inflation factor (VIF). In addition, the mean length of segments defined by participants was compared between groups using a one-way analysis of variance (ANOVA).

Moreover, to assess the similarity in the segmentation between groups at the behavioral level, we calculated each group's segmentation agreements (Bailey et al., 2013). The movie was divided into 1-second bins, and for each participant, we determined whether they identified an event boundary within each bin. Then, in each bin, we calculated the proportion of participants who identified an event boundary here. A higher proportion within a bin indicates greater segmentation agreement. We can assess the similarity between their segmentation patterns by correlating the dynamic changes in these proportions between groups. Additionally, we applied Spearman correlation analysis (Matlab function ‘corr’) between each pair of groups after smoothing the datasets across time points to mitigate the time jitter effect to further evaluate the similarity of the segmentation pattern between the groups. Further, we performed a Fisher Z-transformation on the correlation results to assess whether their differences were statistically significant.

## 3 Results

### 3.1 Behavioral results

**Fig. 2:**
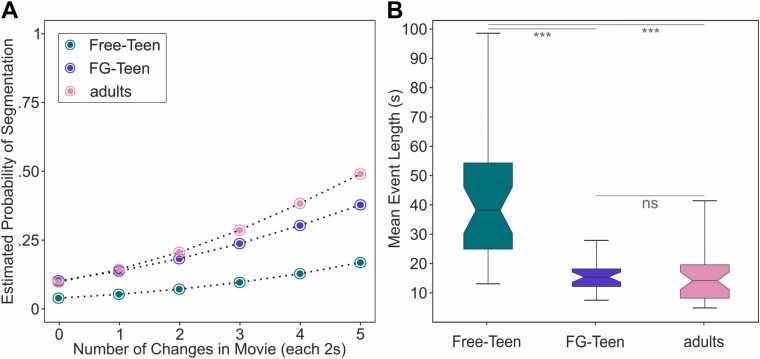
Behavioral results of the mean event length analyses and mixed-effects logistic regression. The Free-Teen group data are shown in green, the FG-Teen group data in purple, and the adult group data in pink. Part A illustrates the predicted likelihood of segmentation (y-axis) as a function of the number of situational changes within a 2 s interval (x-axis) for the three groups. Part B displays the distribution of the mean event length within each group in boxplots. Asterisks denote significant differences between the groups (*** indicates p < .001).

The results of the hierarchical mixed-effects logistic regression for each group pairing are displayed in Fig. 2A and Table 1.

**Table 1:** Results of the post-hoc mixed-effects logistic regression for each two groups.

comparison	intercept	number of changes		group membership		number of changes*group membership	
		coeff	OR with 95 % CI	coeff	OR with 95 % CI	coeff	OR with 95 % CI
Free-Teen [0] vs FG-Teen [1]	−3.20 (p < .001)	.32 (p < .001)	1.38 [1.33–1.43]	1.03 (p < .001)	2.80 [2.23–3.52]	.01 (p = .553)	1.01 [0.97–1.06]
FG-Teen [0] vs adults [1]	−2.17 (p < .001)	.34 (p < .001)	1.40 [1.36–1.44]	−.05 (p = .703)	0.95 [.74–1.23]	.10 (p < .001)	1.11 [1.07–1.15]
Free-Teen [0] vs adults [1]	−3.21 (p < .001)	.32 (p < .001)	1.38 [1.33–1.43]	.99 (p < .001)	2.68 [2.03–3.54]	.12 (p < .001)	1.12 [1.08–1.17]

The number in squared brackets behind the group name in the comparison column indicates the reference group [0] or the comparison group [1]. CI – confidence interval; coeff – coefficient; OR – Odds Ratio.

The main finding concerned the sole effects of the predictor group membership and the interaction effects between the number of changes and the group membership in the comparisons involving the FG-Teen group. When comparing the FG-Teen group with their peers with the free-segmentation instruction, there was a significant effect of the predictor group membership (1.03, p < .001, OR = 2.80, 95 % CI: 2.23–3.52), indicating a higher probability for segmentation behavior in the FG-Teen than in the Free-Teen group. However, the interaction of the predictors number of changes and group membership (.01, p = .553, OR = 1.01, 95 % CI:.97–1.06) did not reach significance, indicating a similar slope of the logistic regression in both groups. In contrast, when comparing the FG-Teen group with the adults, there was no significant effect of the predictor group membership (-.05, p = .703, OR =.95, 95 % CI:.74–1.23), indicating a similar general probability of segmentation behavior in both groups. However, the interaction of the number of changes and the group membership was significant for this comparison (.10, p < .001, OR = 1.11, 95 % CI: 1.07–1.15), indicating a different slope of the logistic regression between both groups.

The comparison of the mean segment length between groups employing an ANOVA revealed a significant difference in the mean segment length between groups (F(2,99) = 43.04, p < .001, η_p_^2^ = 0.47; Fig. 2B). Post-hoc t-tests established significant differences between the Free-Teen and the FG-Teen (t(64) = 6.90, p < .001, Cohen’s d = 1.70) and the adults (t(68) = 6.82, p < .001, Cohen’s d = 1.71), but no significant difference between the adults and the FG-Teen group could be found (t(66) = -.32, p = .748).

**Fig. 3:**
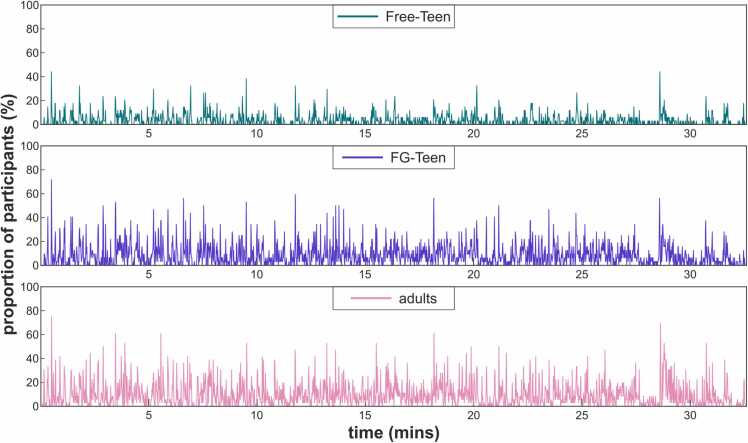
Agreement on the locations of event boundaries across three groups. Each panel displays the proportion of participants (y-axis) from one of the three groups who identified event boundaries at each time point of the movies (x-axis).

Fig. 3 presents the segmentation agreement results for each group. The Spearman correlation analysis of the proportion of participants between each pair of groups yielded the following results: between Free-Teen and FG-Teen, ρ = 0.719 (p < 0.001, df = 1954); between FG-Teen and adults, ρ = 0.742 (p < 0.001, df = 1954); and between Free-Teen and adults, ρ = 0.654 (p < 0.001, df = 1954). Fisher Z-transformation analysis revealed significant differences between ρ = 0.719 and ρ = 0.654 (Z = 3.854, p < 0.001), as well as between ρ = 0.742 and ρ = 0.654 (Z = 5.395, p < 0.001), but no significant difference between ρ = 0.719 and ρ = 0.742 (Z = 1.542, p = 0.123). These findings align with the segmentation patterns shown in Fig. 3, indicating that after the fine-grain instruction, the segmentation pattern of FG-Teen aligns more closely with adults compared to their peers.

### 3.2 MVPA across time

The results of the MVPA across time are shown separately for each group in Supplementary Fig. 1.

In the Free-Teen group, the MVPA established time windows of significant classification ranging from about −203–580 ms relative to the event boundary, with an average AUC of.59 (AUC_min_ =.54, AUC_max_ =.68). In the FG-Teen group, the MVPA established time windows of significant classification ranging from −557–737 ms relative to the event boundary, with an average AUC of.59 (AUC_min_ =.52, AUC_max_ =.71). In the adult group, the MVPA established time windows of significant classification ranging from −527–770 ms relative to the event boundary, with an average AUC of.59 (AUC_min_ =.52, AUC_max_ =.75). The values of the separate significant clusters are displayed in Table 2.

**Table 2:** MVPA results for separate clusters in all three groups.

Group	Cluster	Min time	Max time	Min AUC	Max AUC	Mean AUC
Free-Teen	1	−203 ms	−187 ms	.54	.57	.56
	2	−163 ms	−140 ms	.54	.56	.55
	3	−113 ms	307 ms	.55	.68	.61
	4	317 ms	443 ms	.55	.58	.56
	5	553 ms	580 ms	.54	.56	.55
FG-Teen	1	−557 ms	−540 ms	.52	.53	.53
	2	−410 ms	−360 ms	.52	.54	.53
	3	−350 ms	−327 ms	.52	.54	.53
	4	−320 ms	−303 ms	.53	.53	.53
	5	−290 ms	607 ms	.53	.71	.60
	6	620 ms	737 ms	.52	.54	.53
adults	1	−527 ms	−513 ms	.52	.52	.52
	2	−443 ms	−410 ms	.53	.53	.53
	3	−397 ms	−223 ms	.53	.56	.54
	4	−217 ms	687 ms	.52	.75	.61
	5	693 ms	717 ms	.53	.54	.54
	6	750 ms	770 ms	.52	.54	.53

Min Time – time point of the beginning of the cluster; Max time – time point of the end of the cluster. Time points are given relative to the response of the participant. Min AUC – minimal AUC value in the cluster; Max AUC – maximum AUC value in the cluster; Mean AUC – mean AUC value in the cluster.

**Fig. 4:**
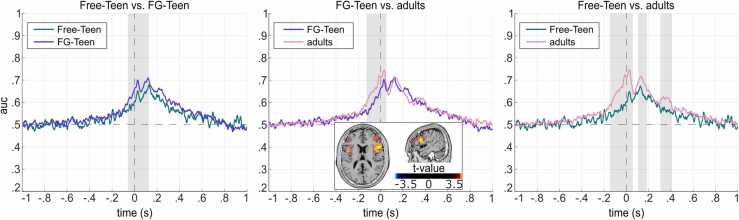
Outcome of MVPA across time for pairwise comparisons. The three panels show the Area Under the Curve (AUC) comparisons between each pair of groups for the across-time MVPA, with grey shadings highlight time periods showing significant differences between the groups. For the comparison of FG-teen vs adults, the significant sLORETA contrast in the time window of significant AUC differences between the groups is presented; color shading indicates t-value.

Importantly, the subsequent comparison of the results revealed significant differences between each two groups (Fig. 4). Comparing the Free-Teen and the FG-Teen, a significantly different time window around the event boundary was found (-60–123 ms, T_sum_ = −130.40, p < .001). Moreover, for the comparison of FG-Teen and adults, a significant difference was revealed around the event boundary (-130–43 ms, T_sum_ = −149.47, p = .006). Also, comparing the Free-Teen and the adults, large time windows with different classification accuracy could be established (first cluster: −147–57 ms, T_sum_ = −244.70, p < .001; second cluster: 107–183 ms, T_sum_ = −53.40, p = .033; third cluster: 307–407 ms, T_sum_ = −75.30, p = .010).

#### 3.2.1 Source localization (sLORETA)

The source localization by means of sLORETA was conducted for each group pairing based on the time windows around the event boundary showing significant differences between these groups in the MVPA across time. For the comparison between FG-Teen and adults (from −130–43 ms), significant differences (t value > 3.46, α = 0.05) were localized in the bilateral inferior frontal gyrus (BA 44, 45 and 9; FG-Teen > adults; Fig. 4B). However, the comparisons between the Free-Teen and FG-Teen groups (from −60–123 ms) and between the Free-Teen and the adult groups (from −147–57 ms) did not yield significant differences.

### 3.3 Temporal generalization MVPA

**Fig. 5:**
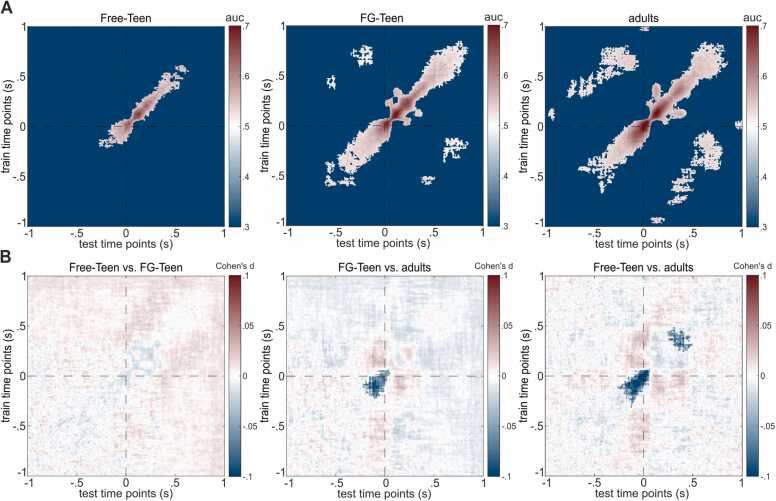
Outcome of temporal generalization MVPA for each group, along with pairwise comparisons. Test time (-1–1 s) on the x-axis and train time (-1–1 s) on the y-axis. Part A illustrates the classification accuracy between Boundary and No-Boundary intervals for each group, with color gradation indicating significant clusters based on the AUC values tested against chance level. Part B shows the effect sizes (Cohen’s d) of comparisons between each pair of groups, with color gradation representing significantly different clusters in time windows of significant classification within the respective groups.

Regarding the temporal generalization MVPA, the comparison results between BI and NBI for each group are displayed in Fig. 5A. For the Free-Teen group, about 3.4 % of the classifications resulted in a significant AUC value, with an average AUC of.57 (AUC_min_ =.51, AUC_max_ =.68) for the significant classifications. These significant classifications were distributed around the diagonal, spanning from approximately −250–600 ms near the boundaries. For the FG-Teen group, about 11.1 % of the classifications resulted in a significant AUC value, with an average AUC of.55 (AUC_min_ =.50, AUC_max_ =.71) for the significant classifications. These significant classifications appeared in two distinct parts: one part was distributed around the diagonal, spanning approximately −600–800 ms near the boundaries, while the other part was completely off-diagonal, ranging from about −250 ms to −100 ms for train time and 600–750 ms for test time. For the adult group, about 15.6 % of the classifications resulted in a significant AUC value, with an average AUC of.55 (AUC_min_ =.51, AUC_max_ =.74) for the significant classifications. These significant classifications also appeared in two distinct parts: the part distributed around the diagonal, similar to the FG-Teen group, spanned approximately −500–800 ms near the boundaries. In contrast, the off-diagonal part was much larger than the FG-Teen group, ranging from about −980 ms to −20 ms for train time and 30–750 ms for test time.

Crucially, we also compared the AUC values between different groups within these significant clusters (Fig. 5B). These analyses revealed significant differences between the FG-Teen and adult groups (FG-Teen < adult) along the diagonal, spanning approximately from −200–50 ms for both train and test times. Even greater differences were found between the Free-Teen and adult groups (Free-Teen < adult), spanning roughly −250–50 ms and 300–500 ms for both train and test times. Lastly, no differences were observed between the Free-Teen and FG-Teen groups.

## 4 Discussion

The current study examined whether and how children, in the process of event segmentation, can exhibit adult-like responses by adjusting their cognitive control style after receiving metacontrol-related instructions. To investigate this question, we presented an event segmentation task to adolescents and adults while concomitantly recording EEG using different instructions. One group of adolescents and a group of adults were asked to segment a movie meaningfully while watching it, whereas another group of adolescents was asked to perform this meaningful segmentation as fine-grainedly as possible. We observed that adolescents instructed to do the segmentation as fine-grained as possible were able to show behavioral and neural responses that were more similar to those of adults compared to their peers. Our findings provide the first evidence that adolescents can partially be more adults-like in event segmentation merely through metacontrol-related instruction, which likely shifts their cognitive control towards a more persistent state.

The behavioral data revealed that adolescents with the “fine-grained” instruction demonstrated more adult-like behavior compared to their peers: adolescents with the “fine-grained” instruction identified more segments than those adolescents who could freely segment the movie (see Fig. 2B). Thus, they performed on a similar level as the adult group. Furthermore, the probability of setting an event boundary given incoming information, such as changes in movie scenes, was higher in the adolescents with the “fine-grained” instruction than in the adolescents without these specific instructions (see Fig. 2B and Table 1), indicating that adolescents with the “fine-grained” instruction were generally more sensitive to these changes compared to their peers. However, compared to adults, adolescents with the “fine-grained” instruction were not increasing their sensitivity with more changes in the movie as much as adults, indicating that adolescents still cannot perform exactly adult-like. Besides, the segmentation pattern of adolescents given the “fine-grained” instruction (see Fig. 3) showed greater similarity to that of adults compared to their peers. Thus, the metacontrol-related instruction partially altered how incoming information was processed: adolescents with “fine-grained” instructions were more focused and restrictive in determining whether the incoming information still fit the current working event model compared to their peers; however, they were still not as proficient as adults when there were multiple changes simultaneously.

Importantly, our neurophysiological findings demonstrated that adolescents with “fine-grained” instructions not only partially showed adult-like behavior in event segmentation behavior, but also changed their neurophysiological. Both across time and temporal generalization MVPA findings (see Supplementary Fig.1 and Fig. 5A) indicated that during the time window around boundaries, neural patterns were distinguishable between BI and NBI for each group (King and Dehaene, 2014), similar to the results of Zhou et al. (Zhou et al., 2024). Subsequent group comparisons revealed that for the MVPA across time, the distinguishability between BI and NBI was greater in adolescents with “fine-grained” instructions compared to their peers who did the free segmentation, yet still lower compared to adults (see Fig. 4). Intriguingly, similar results were obtained for the temporal generalization MVPA (see Fig. 5B). For the comparison between adolescents given "fine-grained" instructions and adults, a significant difference was observed around event boundaries. In comparing adolescents who segmented events freely and adults, significant differences were also found around boundaries, with an additional significant cluster emerging after the boundaries. Better distinguishability implied a clearer differentiation between representations between BI and NBI, which was a fundamental aspect of a persistent cognitive control style (Hommel, 2015b, Hommel and Colzato, 2017a). Of note, the findings indicated an increase of decoding performance toward an adult-like level in adolescents after receiving “fine-grained” instructions, though it remained unclear whether the distinct patterns were similar between the groups. Nevertheless, these findings offered an explanation why adolescents with “fine-grained” instructions were more sensitive to changes in the movie compared to their peers but still not as sensitive as adults in behavioral level. Likely, adolescents with “fine-grained” instructions had shifted their cognitive control style towards a more persistent state characterized by higher levels of top-down cognitive control and a stronger focus on details (Hommel and Colzato, 2017b, Kurby and Zacks, 2008). Consequently, their working event models had become more stringent, leading to establishing an event boundary and the closure of the current event segment in response to even minor deviations from the anticipated future (Richmond et al., 2017, Zacks et al., 2007). Additionally, the off-diagonal clusters observed in the temporal generalization MVPA (Fig. 5 A) indicated the reactivation of neural representations before event boundaries, reflecting the influence and continuity between preceding and current events (Wahlheim and Zacks, 2019, Zhou et al., 2024). Our analysis suggested that this effect was present in adults but absent in adolescents during free segmentation. However, following the “fine-grained” instruction, adolescents also exhibited enhanced reactivation, retrieving more information from recently occurred events. Prospectively, future studies should employ analyses to assess cross-group neural similarity to further clarify how adults and specifically instructed adolescents are different in their mental representations and which similarities they share.

The findings from the source reconstruction within the significant time window in the MVPA across time comparisons between groups provided more insights into the differences in brain activity underlying their different behaviors and neural representations. Heightened bilateral inferior frontal gyrus (IFG, see Fig. 4B) activity modulation among fine-grained adolescents compared to adults might suggest enhanced top-down attentional control within this group, as previous studies had linked IFG activity to top-down attentional control (Braga et al., 2013, Chong et al., 2008, Hampshire et al., 2010). As these adolescents aimed for more fine-grained event segmentation, they required increased focus on movie details, placing greater attentional demands on them. Moreover, they likely differentiated more strongly between occasions with and without event boundaries with respect to their attentional resources compared to adults who did the segmentation freely without additional demands, making the activity modulation larger in the adolescents performing the “fine-grained” instruction. Thus, the stronger IFG activity modulation observed in fine-grained adolescents likely contributed to their shift towards more adult-like event processing. To achieve this, they utilized enhanced top-down attentional control to focus on details, which improved their ability to detect changes in the ongoing movie, leading to more prediction errors and consequently accelerating updates to their working event model and increasing segmentations. However, for the other two group comparisons, the source localization results did not reveal any significant differences. This could be due to the sensitivity of source-level analysis to the timing of the selection. The significant time window observed in MVPA might involve multiple cognitive processes, potentially engaging brain regions associated with each process, which could obscure distinct activity modulations and lead to a lack of detectable differences.

One potential limitation of our study was the influence of motor activity as a confounding factor in MVPA. Although the MVPA successfully distinguished between BI and NBI intervals, it is possible that motor-related responses, in addition to event segmentation, contributed to the observed neural pattern differences within groups. Importantly, our primary group comparison results were unaffected, as this potential confounding factor likely balanced out between groups, supporting the robustness of our main conclusions. Nevertheless, future studies should address this limitation by controlling for motor activity or adopting designs that more effectively disentangle event segmentation from motor responses.

## 5 Conclusion

Overall, our study established a connection between metacontrol and the developmental aspects of event segmentation, demonstrating how metacontrol influences the event segmentation process of adolescents through instruction. Compared to their peers who segmented events freely, adolescents instructed to segment the movie as fine-grained as possible were able to partially be more adult-like in both behavioral and neural decoding performances, potentially by adapting their cognitive control state toward a more persistent mode. This adaptation in fine-grained adolescents likely involved improved top-down attentional control, supported by heightened activity modulations in the inferior frontal gyrus. Therefore, adolescents seem to have some leeway to adapt their behavior to a more mature level.

## Funding

This work was supported by a grant from the Else-Kröner Fresenius Stiftung (Key project) to CB, BH, and VR (2020_EKSE.105) and the Federal Ministry of Education and Research (Bundesministerium für Bildung und Forschung, BMBF) as part of the German Center for Child and Adolescent Health (DZKJ) under the funding code 01GL2405B.

## Data statement

All data can be obtained from the corresponding authors upon request.

## CRediT authorship contribution statement

**Roessner Veit:** Writing – review & editing, Funding acquisition. **Hommel Bernhard:** Writing – review & editing, Funding acquisition. **Prochnow Astrid:** Writing – review & editing, Writing – original draft, Visualization, Supervision, Methodology, Investigation, Conceptualization. **Beste Christian:** Writing – review & editing, Writing – original draft, Supervision, Resources, Methodology, Funding acquisition, Conceptualization. **Zhou Xianzhen:** Writing – review & editing, Writing – original draft, Visualization, Software, Methodology, Formal analysis. **Ghorbani Foroogh:** Writing – original draft, Visualization, Investigation.

## Declaration of Competing Interest

There are no conflicts of interest

## Data Availability

Data will be made available on request.

## References

[bib1] Bailey H.R., Zacks J.M., Hambrick D.Z., Zacks R.T., Head D., Kurby C.A., Sargent J.Q. (2013). Medial temporal lobe volume predicts elders’ everyday memory. Psychol. Sci..

[bib2] Benear S.L., Popal H.S., Zheng Y., Tanrıverdi B., Murty V.P., Perlman S.B., Olson I.R., Newcombe N.S. (2023). Setting boundaries: development of neural and behavioral event cognition in early childhood. Dev. Sci..

[bib3] Braga R.M., Wilson L.R., Sharp D.J., Wise R.J.S., Leech R. (2013). Separable networks for top-down attention to auditory non-spatial and visuospatial modalities. NeuroImage.

[bib4] Carter C.S., Braver T.S., Barch D.M., Botvinick M.M., Noll D., Cohen J.D. (1998). Anterior cingulate cortex, error detection, and the online monitoring of performance. Science.

[bib5] Chai X., Tang L., Gabrieli J., Ofen N. (2024). From vision to memory: how scene-sensitive regions support episodic memory formation during child development. Dev. Cogn. Neurosci..

[bib6] Chong T.T.-J., Williams M.A., Cunnington R., Mattingley J.B. (2008). Selective attention modulates inferior frontal gyrus activity during action observation. NeuroImage.

[bib7] Cowan N. (2016). Working memory maturation: can we get at the essence of cognitive growth?. Perspect. Psychol. Sci..

[bib8] Delorme A., Makeig S. (2004). EEGLAB: an open source toolbox for analysis of single-trial EEG dynamics including independent component analysis. J. Neurosci. Methods.

[bib9] Dippel G., Beste C. (2015). A causal role of the right inferior frontal cortex in implementing strategies for multi-component behaviour. Nat. Commun..

[bib10] Fahrenfort J.J., van Driel J., van Gaal S., Olivers C.N.L. (2018). From ERPs to MVPA using the amsterdam decoding and modeling toolbox (ADAM). Front. Neurosci..

[bib11] Gerber, E., 2022. permutest - File Exchange - MATLAB Central [WWW Document]. URL https://ww2.mathworks.cn/matlabcentral/fileexchange/71737-permutest (accessed 7.15.24).

[bib12] Ghin F., Eggert E., Gholamipourbarogh N., Talebi N., Beste C. (2024). Response stopping under conflict: the integrative role of representational dynamics associated with the insular cortex. Hum. Brain Mapp..

[bib13] Ghorbani F., Zhou X., Talebi N., Roessner V., Hommel B., Prochnow A., Beste C. (2024). Neural connectivity patterns explain why adolescents perceive the world as moving slow. Commun. Biol..

[bib14] Gogolla N. (2017). The insular cortex. Curr. Biol..

[bib15] Graf K., Jamous R., Mückschel M., Bluschke A., Beste C. (2024). Delayed modulation of alpha band activity increases response inhibition deficits in adolescents with AD(H)D. NeuroImage: Clin..

[bib16] Grootswagers T., Wardle S.G., Carlson T.A. (2017). Decoding dynamic brain patterns from evoked responses: a tutorial on multivariate pattern analysis applied to time series neuroimaging data. J. Cogn. Neurosci..

[bib17] Ham T., Leff A., de Boissezon X., Joffe A., Sharp D.J. (2013). Cognitive control and the salience network: an investigation of error processing and effective connectivity. J. Neurosci..

[bib18] Hampshire A., Chamberlain S.R., Monti M.M., Duncan J., Owen A.M. (2010). The role of the right inferior frontal gyrus: inhibition and attentional control. NeuroImage.

[bib19] Hommel B., Elliot A.J. (2015). Advances in Motivation Science.

[bib20] Hommel B. (2015). Advances in Motivation Science.

[bib21] Hommel B., Colzato L., Beste C. (2024). No convincing evidence for the independence of persistence and flexibility. Nat. Rev. Psychol..

[bib22] Hommel B., Colzato L.S. (2017). Meditation and metacontrol. J. Cogn. Enhanc..

[bib23] Hommel B., Colzato L.S. (2017). The social transmission of metacontrol policies: mechanisms underlying the interpersonal transfer of persistence and flexibility. Neurosci. Biobehav. Rev..

[bib24] King J.-R., Dehaene S. (2014). Characterizing the dynamics of mental representations: the temporal generalization method. Trends Cogn. Sci..

[bib25] Koechlin E. (2014). An evolutionary computational theory of prefrontal executive function in decision-making. Philos. Trans. R. Soc. B.

[bib26] Kurby C.A., Zacks J.M. (2008). Segmentation in the perception and memory of events. Trends Cogn. Sci..

[bib27] Le ballon rouge, 1956. Films Montsouris.

[bib28] Levine D., Buchsbaum D., Hirsh-Pasek K., Golinkoff R.M. (2019). Finding events in a continuous world: a developmental account. Dev. Psychobiol..

[bib29] Mansouri F.A., Koechlin E., Rosa M.G.P., Buckley M.J. (2017). Managing competing goals — a key role for the frontopolar cortex. Nat. Rev. Neurosci..

[bib30] Meyer M., Baldwin D.A. (2011). Assessing young children’s hierarchical action segmentation. Proc. Annu. Meet. Cogn. Sci. Soc..

[bib31] Mullen T., Kothe C., Chi Y.M., Ojeda A., Kerth T., Makeig S., Cauwenberghs G., Jung T.-P. (2013). Real-time modeling and 3D visualization of source dynamics and connectivity using wearable EEG. Annu Int Conf. IEEE Eng. Med Biol. Soc. 2013.

[bib32] Ocklenburg S., Friedrich P., Fraenz C., Schlüter C., Beste C., Güntürkün O., Genç E. (2018). Neurite architecture of the planum temporale predicts neurophysiological processing of auditory speech. Sci. Adv..

[bib33] Oostenveld R., Fries P., Maris E., Schoffelen J.-M. (2011). 2010. FieldTrip: open source software for advanced analysis of MEG, EEG, and invasive electrophysiological data. Comput. Intell. Neurosci..

[bib34] Parra L.C., Spence C.D., Gerson A.D., Sajda P. (2005). Recipes for the linear analysis of EEG. Neuroimage.

[bib35] Pascual-Marqui R.D. (2002). Standardized low-resolution brain electromagnetic tomography (sLORETA): technical details. Methods Find. Exp. Clin. Pharm..

[bib36] Pedroni A., Bahreini A., Langer N. (2019). Automagic: standardized preprocessing of big EEG data. Neuroimage.

[bib37] Petruo V., Takacs A., Mückschel M., Hommel B., Beste C. (2021). Multi-level decoding of task sets in neurophysiological data during cognitive flexibility. iScience.

[bib38] Prochnow A., Mückschel M., Eggert E., Senftleben J., Frings C., Münchau A., Roessner V., Bluschke A., Beste C. (2024). The ability to voluntarily regulate theta band activity affects how pharmacological manipulation of the catecholaminergic system impacts cognitive control. Int. J. Neuropsychopharmacol..

[bib39] Prochnow A., Zhou X., Ghorbani F., Wendiggensen P., Roessner V., Hommel B., Beste C. (2024). The temporal dynamics of how the brain structures natural scenes. Cortex.

[bib40] Prochnow A., Zhou X., Ghorbani F., Roessner V., Hommel B., Beste C. (2024). Event segmentation in ADHD: neglect of social information and deviant theta activity point to a mechanism underlying ADHD. Gen. Psych..

[bib41] Radvansky G.A., Copeland D.E. (2006). Memory retrieval and interference: working memory issues. J. Mem. Lang..

[bib42] Radvansky G.A., Zacks J.M. (2014).

[bib43] Ren J., Wharton-Shukster E., Bauer A., Duncan K., Finn A.S. (2021). Events structure information accessibility less in children than adults. Cognition.

[bib44] Renton A.I., Painter D.R., Mattingley J.B. (2022). Optimising the classification of feature-based attention in frequency-tagged electroencephalography data. Sci. Data.

[bib45] Richmond L.L., Gold D.A., Zacks J.M. (2017). Event perception: translations and applications. J. Appl. Res. Mem. Cogn..

[bib46] Sargent J.Q., Zacks J.M., Hambrick D.Z., Zacks R.T., Kurby C.A., Bailey H.R., Eisenberg M.L., Beck T.M. (2013). Event segmentation ability uniquely predicts event memory. Cognition.

[bib47] Sekihara K., Sahani M., Nagarajan S.S. (2005). Localization bias and spatial resolution of adaptive and non-adaptive spatial filters for MEG source reconstruction. Neuroimage.

[bib48] Spencer J.P. (2020). The development of working memory. Curr. Dir. Psychol. Sci..

[bib49] Takacs A., Mückschel M., Roessner V., Beste C. (2020). Decoding stimulus-response representations and their stability using EEG-based multivariate pattern analysis. Cereb. Cortex Commun..

[bib50] Treder M.S. (2020). MVPA-Light: a classification and regression toolbox for multi-dimensional data. Front. Neurosci..

[bib51] Van Schependom J., Baetens K., Nagels G., Olmi S., Beste C. (2024). Neurophysiological avenues to better conceptualizing adaptive cognition. Commun. Biol..

[bib52] Wahlheim C.N., Zacks J.M. (2019). Memory guides the processing of event changes for older and younger adults. J. Exp. Psychol. Gen..

[bib53] Wang X., Talebi N., Zhou X., Hommel B., Beste C. (2024). Neurophysiological dynamics of metacontrol states: EEG insights into conflict regulation. NeuroImage.

[bib54] Widmann A., Schröger E., Maess B. (2015). Digital filter design for electrophysiological data-a practical approach. J. Neurosci. Methods.

[bib55] Winkler I., Brandl S., Horn F., Waldburger E., Allefeld C., Tangermann M. (2014). Robust artifactual independent component classification for BCI practitioners. J. Neural Eng..

[bib56] Yates, T.S., Skalaban, L.J., Ellis, C.T., Bracher, A.J., Turk-Browne, N.B., 2022. Neural event segmentation of continuous experience in human infants. 10.1073/pnas.2200257119 PMC9618143 36252007

[bib57] Yu S., Stock A.-K., Münchau A., Frings C., Beste C. (2023). Neurophysiological principles of inhibitory control processes during cognitive flexibility. Cereb. Cortex.

[bib58] Zacks J.M. (2010). The brain’s cutting-room floor: segmentation of narrative cinema. Front. Hum. Neurosci..

[bib59] Zacks J.M. (2019). Event perception and memory. Annu. Rev..

[bib60] Zacks J.M., Braver T.S., Sheridan M.A., Donaldson D.I., Snyder A.Z., Ollinger J.M., Buckner R.L., Raichle M.E. (2001). Human brain activity time-locked to perceptual event boundaries. Nat. Neurosci..

[bib61] Zacks J.M., Speer N.K., Reynolds J.R. (2009). Segmentation in reading and film comprehension. J. Exp. Psychol. Gen..

[bib62] Zacks J.M., Speer N.K., Swallow K.M., Braver T.S., Reynolds J.R. (2007). Event perception: a mind-brain perspective. Psychol. Bull..

[bib63] Zacks J.M., Swallow K.M. (2007). Event segmentation. Curr. Dir. Psychol. Sci..

[bib64] Zacks J.M., Tversky B. (2001). Event structure in perception and conception. Psychol. Bull..

[bib65] Zhou X., Ghorbani F., Roessner V., Hommel B., Prochnow A., Beste C. (2024). The metacontrol of event segmentation—a neurophysiological and behavioral perspective. Hum. Brain Mapp..

