# Supplementary material for: Metacontrol instructions lead to adult-like event segmentation in adolescents
Source: Dev Cogn Neurosci. 2025 Jan 30;72:101521. doi: 10.1016/j.dcn.2025.101521 (PMC11833649; doi:10.1016/j.dcn.2025.101521)
Supplement: Supplementary file 1 — Supplementary material [file mmc1.docx]

**SUPPLEMENTAL MATERIAL**

**Metacontrol Instructions Lead to Adult-Like Event Segmentation in Adolescents**


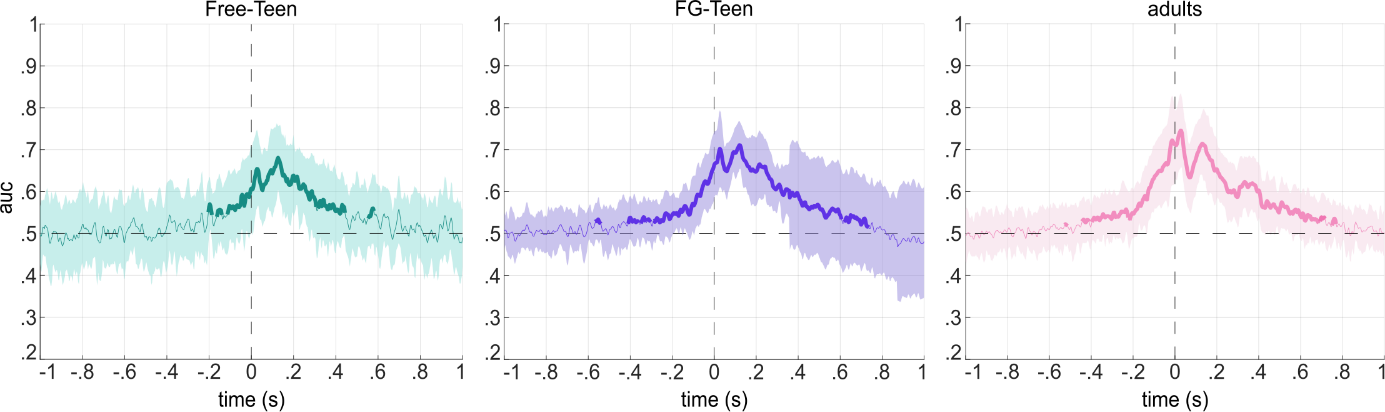


*Supplementary Figure 1 - Outcome of MVPA across time for each group.*

The three panels illustrate the Area Under the Curve (AUC) for each group, with bold lines indicating statistically significant classifications between Boundary and No-Boundary intervals above chance level. The shading around the lines represents the standard deviation of the AUC across the sample.
